# The QUEST for quality online health information: validation of a short quantitative tool

**DOI:** 10.1186/s12911-018-0668-9

**Published:** 2018-10-19

**Authors:** Julie M. Robillard, Jessica H. Jun, Jen-Ai Lai, Tanya L. Feng

**Affiliations:** 1 Division of Neurology, Department of Medicine, The University of British Columbia, Vancouver, Canada; 2 BC Children’s & Women’s Hospital, Vancouver, Canada; 3 Djavad Mowafaghian Centre for Brain Health, The University of British Columbia, 2215 Wesbrook Mall, Room 3450D, Vancouver, BC V6T 2B5 Canada

**Keywords:** Online health information, Quality evaluation, eHealth, Instrument validation

## Abstract

**Background:**

Online health information is unregulated and can be of highly variable quality. There is currently no singular quantitative tool that has undergone a validation process, can be used for a broad range of health information, and strikes a balance between ease of use, concision and comprehensiveness. To address this gap, we developed the QUality Evaluation Scoring Tool (QUEST). Here we report on the analysis of the reliability and validity of the QUEST in assessing the quality of online health information.

**Methods:**

The QUEST and three existing tools designed to measure the quality of online health information were applied to two randomized samples of articles containing information about the treatment (*n* = 16) and prevention (*n* = 29) of Alzheimer disease as a sample health condition. Inter-rater reliability was assessed using a weighted Cohen’s kappa (κ) for each item of the QUEST. To compare the quality scores generated by each pair of tools, convergent validity was measured using Kendall’s tau (τ) ranked correlation.

**Results:**

The QUEST demonstrated high levels of inter-rater reliability for the seven quality items included in the tool (κ ranging from 0.7387 to 1.0, *P* < .05). The tool was also found to demonstrate high convergent validity. For both treatment- and prevention-related articles, all six pairs of tests exhibited a strong correlation between the tools (τ ranging from 0.41 to 0.65, *P* < .05).

**Conclusions:**

Our findings support the QUEST as a reliable and valid tool to evaluate online articles about health. Results provide evidence that the QUEST integrates the strengths of existing tools and evaluates quality with equal efficacy using a concise, seven-item questionnaire. The QUEST can serve as a rapid, effective, and accessible method of appraising the quality of online health information for researchers and clinicians alike.

**Electronic supplementary material:**

The online version of this article (10.1186/s12911-018-0668-9) contains supplementary material, which is available to authorized users.

## Background

The Internet has revolutionized how information is distributed and has led to the rapid expansion of health resources from a wide variety of content providers, ranging from government organizations to for-profit companies. Consulting online health information is an increasingly popular behavior, with 80% of Internet users engaging in this activity [1]. Health information consumers worldwide, particularly those in developing countries and remote areas, may benefit from accessible and immediate retrieval of up-to-date information [2, 3]. This new information gateway also promotes autonomy by allowing patients to be more active in their health [4].

The dynamic nature of the Internet, however, introduces important concerns in parallel with these benefits. Online information is unregulated and can be of highly variable quality [5]. This has critical implications for users as it is estimated that over half of the adult population in the United States and Canada does not possess an adequate level of health literacy [6, 7], and low health literacy is negatively correlated with the ability to discriminate between high and low quality eHealth information [8]. Compounding this issue, there is a growing number of individuals who use online information to guide health care decisions, either for themselves or on behalf of another person. It is therefore crucial to develop effective methods to evaluate online health information [9]. To this end, there have been many efforts to develop tools that assess the quality of online health information; while such tools will not solve the issue of regulation, they can assist end-users, health care professionals and researchers in differentiating between high- and low-quality online sources.

A scoping review of the literature on the evaluation of health information was conducted using Arksey and O’Malley’s six-stage methodological framework [10]. The scoping review aimed to identify existing health information evaluation tools and information available in the literature on their demonstrated validity and reliability. An iterative team approach was used to determine a search strategy balancing feasibility and comprehensiveness. Data was collected via keyword searches and citation searches on Google Scholar and PubMed. Seven combinations of following keywords were used: online, health information, evaluate, evaluation, tool, quality, validity, testing, validation, and assessment. A total of 49 records were retrieved between January 15, 2016 and February 5, 2016. Thirty-six^1^ of these articles were included in the review based on the following inclusion criteria: 1) the article is in the English language; 2) validation of an assessment tool related to quality of health information was the focus of the article. Fifteen tools^2^ currently available in the literature were identified in the scoping review. A follow-up search was conducted on September 10, 2018, yielding three additional tools: the Quality Index for health-related Media Reports (QIMR) [11], the “Date, Author, References, Type, and Sponsor” (DARTS) tool [12] and Index of Scientific Quality (ISQ) [13]. The tools identified range from generic assessments, intended for use across multiple domains of online health information, to assessments targeted to a specific: 1) health condition [14, 15]; 2) aspect of a condition such as treatment [12, 16]; 3) audience [17, 18]; or 4) type of media [11, 13]. As such, a disadvantage of existing tools is that they are limited in the scope of their application.

Many of the existing tools identified, with some notable exceptions, are lengthy and potentially arduous to use, out-dated, or no longer available online [3]. Some tools consist of sets of criteria or checklists that do not provide a quantitative result, making it difficult to compare information from different sources. Finally, while there are many studies evaluating online health information using existing quality evaluation tools, studies assessing the validity, reliability, and efficacy of the tools themselves are lacking in the medical informatics literature.

At present, there is no clear universal standard for evaluating the quality of online health information [3]. Many researchers and regulatory bodies, including the World Health Organization, have called for the establishment of such a standard [9]. Quality criteria across existing tools often overlap and thus may serve as the basis for developing a universalized set of criteria. Aslani et al. distilled a total of 34 criteria from five evaluation tools into 10 general criteria, subdivided into four categories: author, sponsors, and individual(s) responsible for the website; purpose of the website and supporting evidence; design, ease of use, privacy, and interactibility of the website; and date of update [19]. These aggregate criteria largely correspond to groupings of criteria generated in previous reviews of the literature [20, 21]. The criteria also align with the “5 C’s” of website quality (credibility, currency, content, construction, and clarity) outlined by Roberts [22].

Of the many criteria-based assessment tools that have been developed, only a fraction have been tested for inter-rater reliability and even fewer have been validated [23]. Of tools that have reported measuring inter-rater reliability, few have consistently achieved acceptable levels of agreement across all criteria [24]. Gagliardi and Jadad [25] found that only five of 51 rating instruments they evaluated provided explicit evaluation criteria and none were validated. In a more recent review of 12 instruments by Breckons et al. [23], only two tools, DISCERN and the LIDA Minervation tool, contained any measure of reliability and validity. The DISCERN tool is the only tool currently available online for which substantive validation data is publicly available. During development of the tool, a questionnaire administered to information providers and self-help organizations was used to establish face and content validity and inter-rater reliability [16]. Additionally, external assessments indicated significant correlation with content coverage and correctness [26], good internal consistency, and significant inter-rater reliability [27]. Past comparisons to other tools, including the Mitretek Information Quality Tool (IQT) [27], Sandvik quality scale [28], EQIP [17], and DARTS [26], found significant convergent validity with DISCERN. However, DISCERN is limited in its scope of application as it is focused on treatment information and as such is not applicable to online content about other aspects of health and illness including prevention and diagnosis.

**Fig. 1:**
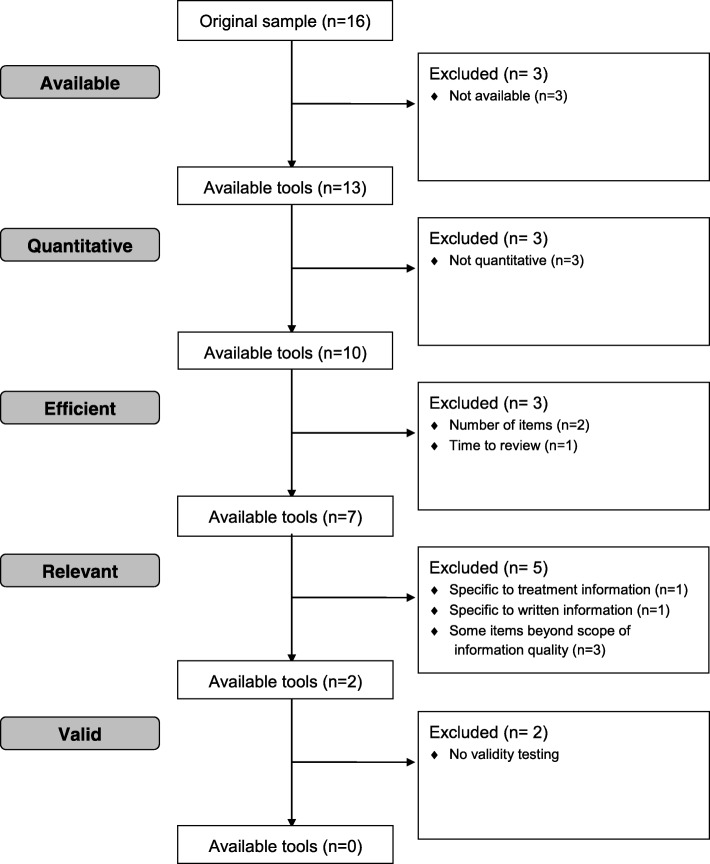
Review of existing quality evaluation tools (*n* = 16). Adapted from the CONSORT 2010 Flow Diagram available at http://www.consort-statement.org/consort-statement/flow-diagram

**Fig. 2:**
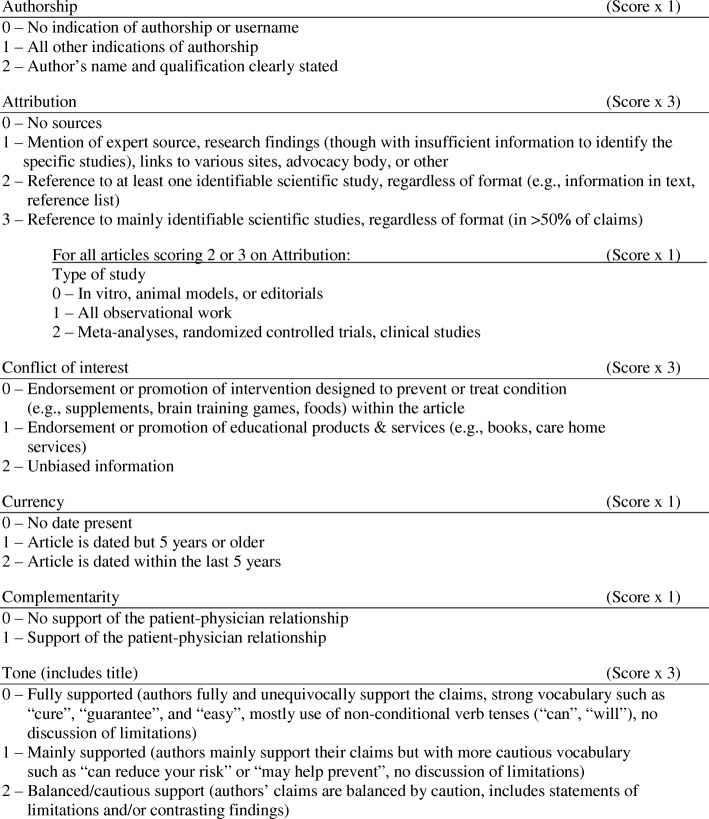
Description of the QUEST criteria. Scores in the individual sections are weighted and summed to generate a total score of up to 28

There is currently no singular quantitative tool that has undergone a validation process, can be used for a broad range of health information, and strikes a balance between ease of use, concision and comprehensiveness (Fig. 1). To address these gaps, we developed the QUality Evaluation Scoring Tool (QUEST). The QUEST quantitatively measures six aspects of the quality of online health information: authorship, attribution, conflict of interest, currency, complementarity, and tone (Fig. 2), yielding an overall quality score between 0 and 28. Attribution is measured through two items, yielding a seven-item evaluation for six measures of health information quality. The criteria were chosen based on a review of existing tools used to evaluate the quality of online information by Chumber et al. [29], Sandvik et al. [28], and Silberg et al. [30]; content analysis was used to capture the overarching categories assessed by these tools [31].

When applying the QUEST, each of the seven quality items is assigned a weighted score. The weighting of each criterion was developed based on two factors: (i) how critical it is to the overall quality of the article, established by a preliminary analysis of a sample of websites, and (ii) consideration of the criterion’s ethical implications. One criterion, attribution, is measured through a two-step process by identifying (1) the presence of references to scientific studies and, (2) the type of studies referenced, if any (e.g., animal models, observational studies, meta-analyses, clinical trials). The second item, which assigns a ranking based on the types of studies included, is in accordance with the GRADE criteria for clinical evidence [32]. This item is scored as a support to the overall quality of the health information presented, not as a judgment of the referenced studies’ quality.

The aim of the present study was to evaluate whether the QUEST reliably measures a similar concept of quality to existing tools. Here we present the results of the inter-rater reliability and convergent validity analyses.

## Methods

### Sample

For the purposes of this study, Alzheimer disease (AD) was used as the reference health condition as there is an abundance of online articles on this topic [33, 34], and there are established methodologies for sampling in this field [31]. Online articles containing AD treatment information were retrieved using a location-disabled search on Google.com/ncr (no country redirect) to avoid localized results. Searches were conducted on an application that prevents the collection of browsing history and cookies during the search and browsing history and cookies were cleared before each search to ensure that search results were not influenced by these factors. Forty-eight different combinations of search terms related to the treatment of AD were used. Articles were extracted from the first three pages of search results, based on analyses of aggregate data on online activity patterns indicating that most Internet users tend not to view past the third page of search results [35]. Each page of search results was comprised of nine articles, totalling 27 articles for each key word combination. Inclusion criteria for the articles were: 1) the article is in the English language; 2) no payment or login is required to access the article; 3) treatment of AD is the main focus of the article as determined by the content of the headline and lead paragraph; and 4) treatment interventions discussed in the article are not solely based on animal experiments. An automatic number generator was used to obtain random 10% samples of articles that met these inclusion criteria in this present study.

In a separate sample, online articles containing information about the prevention of AD were retrieved using similar methods. To retrieve these articles, 105 combinations of search terms related to AD prevention were used. Articles were screened according to criteria 1, 2, and 3 of the inclusion criteria used for treatment articles, with the exception that criterion 3 focused on prevention rather than treatment. As with the treatment-related articles, a random 10% sample of relevant articles was used for validation. In the present study, an article is defined as the heading on a webpage and the text associated with it, excluding links, images and advertising outside of the main body of text. We selected this sampling strategy based on previous investigations of inter-rater reliability and validity of similar tools that have assessed samples of 12 to 40 websites [23, 26, 27, 36, 37].

### Reliability analysis

The QUEST was applied to each sample of online articles by two independent raters (JJ and TF for the prevention sample and JJ and JL for the treatment sample). Two of the three raters were naïve to tool development. To evaluate inter-rater agreement between the two reviewers, a weighted Cohen’s kappa (κ) was calculated for each item of the tool. Agreement was interpreted according to Landis and Koch, where a κ-value of 0.0 to 0.2 indicates slight agreement, 0.21 to 0.40 indicates fair agreement, 0.41 to 0.60 indicates moderate agreement, 0.61 to 0.80 indicates substantial agreement, and 0.81 to 1.0 indicates almost perfect or perfect agreement [38]. Following initial ratings of the samples, remaining disagreements were resolved by discussion to achieve 100% agreement.

### Validity analysis

Three tools were selected for comparison with the QUEST based on availability, ubiquity of use, and relatedness of quality criteria and were applied to both samples. The Health on the Net Foundation’s HONcode Code of Conduct and the DISCERN instrument [16] are two of the most widely used and cited quality evaluation tools [5]. The DISCERN instrument is a 16-item questionnaire intended specifically for evaluation of health information on treatment choices, and has been found to demonstrate good inter-rater reliability and face and content validity. The HONcode Code of Conduct is a set of eight criteria used to certify websites containing health information [5]; its creators also developed a Health Website Evaluation Tool, which was used in this analysis due to its closer similarity in purpose and format to the QUEST and other tools. General quality items developed by Sandvik comprised the final tool for comparison [28]. All three tools selected for comparison are criteria-based, can be applied by a non-expert user, and contain quality criteria that, in general, align categorically with each other and the QUEST (Table 1).

**Table 1:** Comparison of quality items used in the QUEST, HONcode, Sandvik, and DISCERN tools

Quality criteria	QUEST	HONcode	Sandvik	DISCERN
Attribution	X	X	X	X
Currency	X	X	X	X
Authorship	X	X	X	
Balance		X	X	X
Reliability		X		X
Interactivity		X	X	
Tone	X			
Conflict of interest	X			
Complementarity	X			
Mission/target		X		
Audience		X		
Privacy		X		
Overall Reliability		X		
Ownership			X	
Navigability			X	
Quality of information on treatment choices				X
Overall Rating				X

The QUEST and the three tools for comparison were applied to the 10% sample of treatment-related articles and the 10% sample of prevention-related articles by one investigator. The numeric scores obtained by each tool were converted to percentage scores to facilitate comparison across tools. The distribution of quality scores generated by the QUEST was plotted as a histogram to determine whether a spectrum of quality was captured by the sample (see Fig. 1, Robillard and Feng 2017 [31]).

For each tool, the articles were ranked based on their scores and rankings were compared across tools in order to measure convergence. To accomplish this, a two-tailed Kendall’s tau (τ) ranked correlation [39] was used to measure convergence at α = .05. Confidence intervals (CI) of 95% for τ were calculated using Z_0.05_. Six correlational tests, each comparing a unique pair of tools, were performed to compare the results of the QUEST, HONcode, Sandvik, and DISCERN tools. This process was carried out for both the samples of treatment- and prevention-related articles.

## Results

### Sample

A total of 496 treatment articles were retrieved, with 163 of the articles meeting criteria for inclusion in the analysis and the random 10% sample consisted of 16 articles (Additional file 1). Similarly, a sample of 308 prevention articles were collected, 296 of which met inclusion criteria and 29 articles were included in the random 10% sample (Additional file 2). These articles were analyzed using the QUEST in previous quality analysis studies of articles about the prevention of AD [31]. The scores generated by each of the tools for the treatment and prevention samples are included in additional files [see Additional files 1 and 2].

### Reliability analysis

#### Treatment

The level of inter-rater reliability was substantial between the reviewers for Attribution (κ = 0.79), high to near perfect for authorship, currency, complementarity and tone (κ ranging from 0.86 to 0.91), and perfect for type of study and conflict of interest (Table 2).

**Table 2:** Weighted Cohen’s kappa, standard error and 95% CI for treatment articles (*n* = 16)

	Authorship	Attribution	Type of study	Conflict of interest	Currency	Complementarity	Tone
Observed kappa	0.91	0.79	1	1	0.86	0.86	0.91
SE	0.08	0.10	0	0.24	0.13	0.13	0.08
95% CI	0.75, 1	0.58, 0.99	1, 1	0.32, 1	0.60, 1	0.60, 1	0.75, 1

#### Prevention

Inter-rater reliability between the two reviewers ranged from substantial to perfect agreement for each of the seven items included in the QUEST (κ ranging from 0.74 to 1.0; Table 3).

**Table 3:** Weighted Cohen’s kappa, standard error and 95% CI for prevention articles (*n* = 29)

	Authorship	Attribution	Type of study	Conflict of interest	Currency	Complementarity	Tone
Observed kappa	0.88	0.89	0.89	0.74	1	0.75	0.95
SE	0.09	0.06	0.06	0.16	0	0.14	0.04
95% CI	0.71, 1	0.78, 1	0.77, 1	0.43, 1	1, 1	0.49, 1	0.86, 1

### Validity analysis

#### Treatment

Scores obtained using HONcode had the widest range, 15–100%. Scores obtained using the Sandvik criteria had a narrower range, 43–100%. The DISCERN instrument returned the narrowest range of scores, 45–86%. The QUEST generated a range of scores (25–100%) wider than those generated by both the DISCERN tool and Sandvik criteria, but narrower than that of HONcode.

The median percentage scores returned by the DISCERN and HONcode tools were 59% and 62% respectively, while the Sandvik criteria generated a median score of 86%. Again, the median score generated by the QUEST, 71%, fell between those of the other instruments.

Quality analysis of the prevention-related articles generated similar results. HONcode generated the widest range of scores (22–100%), while DISCERN returned the narrowest range (30–88%). The range of scores obtained using the Sandvik criteria (29–93%) fell between the ranges generated by the HONcode and DISCERN instruments. The QUEST generated a range of scores (29–96%) wider than those of DISCERN and Sandvik, but narrower than that of HONcode.

On the lower end, the median percentage score obtained using the DISCERN criteria was 54%. On the upper end, the median score generated by HONcode was 68%. Between these values, both the Sandvik criteria and the QUEST returned a median score of 64%.

Of the six correlational tests performed between unique pairs of tools on the articles related to treatment, all six tests demonstrated a significant correlation between the tools (Table 4). Values of τ ranged from 0.47 (QUEST and HONcode) and 0.53 (HONcode and Sandvik) on the lower end to 0.62 (QUEST and Sandvik) and 0.65 (QUEST and DISCERN) on the higher end (*P* < .05 for all tests).

**Table 4:** Kendall’s tau, standard error, 95% CI, and *P*-value of each test for treatment articles (*n* = 16)

	Kendall’s tau (95% CI)	SE	*P*-value
QUEST vs HONcode	0.47 (0.09–0.85)	0.19	0.015
QUEST vs Sandvik	0.62 (0.23–1.01)	0.20	0.002
QUEST vs DISCERN	0.65 (0.28–1.02)	0.19	< 0.001
HONcode vs Sandvik	0.53 (0.13–0.92)	0.20	0.009
HONcode vs DISCERN	0.58 (0.20–0.96)	0.19	0.003
Sandvik vs DISCERN	0.58 (0.19–0.96)	0.20	0.004

#### Prevention

Similarly, all six correlational tests performed on the prevention sample demonstrated a significant correlation between the tools (*P* < .05; Table 5). The weakest correlations were found between Sandvik and DISCERN, and the QUEST and DISCERN, which produced τ- values of 0.41 and 0.55 respectively. The strongest correlations were found between the QUEST and Sandvik (τ =0.62) and the QUEST and HONcode (τ = 0.64).

**Table 5:** Kendall’s tau, standard error, 95% CI, and *P*-value of each test for prevention articles (*n* = 29)

	Kendall’s tau (95% CI)	SE	*P*-value
QUEST vs HONcode	0.64 (0.37–0.99)	0.14	< 0.001
QUEST vs Sandvik	0.62 (0.34–0.90)	0.14	< 0.001
QUEST vs DISCERN	0.55 (0.29–0.82)	0.14	< 0.001
HONcode vs Sandvik	0.61 (0.33–0.89)	0.14	< 0.001
HONcode vs DISCERN	0.57 (0.31–0.84)	0.14	< 0.001
Sandvik vs DISCERN	0.41 (0.13–0.68)	0.14	0.004

## Discussion

In the present study to validate a novel tool to assess the quality of health information available on the Internet, we find the QUEST to have high inter-rater reliability and convergent validity when applied to two samples of online articles on AD. The results of the validity analysis of treatment and prevention samples indicate that the rankings of quality scores generated by the QUEST converge with those generated by three other tools – the HONcode Health Website Evaluation tool, the DISCERN instrument, and the Sandvik criteria.

For the sample of articles on AD treatment, the strong correlation between the QUEST and the DISCERN instrument suggests that these tools evaluate a similar concept of quality. As past findings indicate that the DISCERN tool is itself a valid tool for assessing treatment information, its high level of convergence with the QUEST confers promising preliminary evidence for the validity of the QUEST. One limitation of the DISCERN tool is the ambiguity in applying a Likert scale to the data. The QUEST addresses this limitation by providing specific descriptions of the criteria for each possible score for a given item.

The QUEST’s lower level of convergence with the HONcode’s evaluation of treatment-related articles may indicate a wider gap between interpretations of the concept of quality evaluated by these two tools. The HONcode tool places emphasis on aspects that are not assessed by the QUEST, such as the website’s mission, target audience, privacy policy, and interactivity [40], all of which expand on the concept of quality but increase the time required to apply the tool. However, there may be other factors that account for the discrepancy between the tools’ rankings. There exist some ambiguities in scoring websites using HONcode that are intrinsic to the design of the tool. For example, with a few exceptions, the HONcode rates questions on a dichotomous scale (Yes/No). This rating system, unlike the Likert-type scales used by the QUEST, DISCERN, and Sandvik [28], does not allow for an assessment beyond an absence or presence of criteria. Finally, some criteria are only marginally or not applicable to many websites’ content. For example, one question asks the responder to evaluate banner content, and website design has moved away from these types of site elements.

Analysis of the scores generated from the sample of prevention-related articles found the strongest correlation between the QUEST and HONcode. Conversely, the QUEST displayed the poorest convergence with the DISCERN instrument. The discrepancy between these findings and those from the treatment sample, which found the strongest convergence between the QUEST and DISCERN and the weakest between the QUEST and HONcode, may reflect intrinsic differences in the purpose of the tools. The DISCERN instrument was developed specifically for the quality evaluation of treatment information, whereas the QUEST, HONcode and Sandvik criteria were developed for health information more broadly.

Overall findings demonstrate a high degree of inter-rater reliability for all seven items of the QUEST. In their evaluation of the DISCERN instrument, Charnock et al. [16] found that lower agreement scores were generally associated with criteria that required more subjective assessment, such as ratings about areas of uncertainty or questions requiring scaled responses. Results from the current study indicate that more subjective items in the QUEST, such as attribution, conflict of interest and tone, achieve about equal or higher levels of inter-reliability as more objective items. Results from the reliability analysis suggest that the QUEST criteria may serve as an effective framework for current as well as future iterations of quality evaluation resources.

The QUEST offers three main advantages over existing tools. Foremost, the QUEST condenses a wide range of quality evaluation criteria into a brief, seven-item questionnaire that evaluates quality with comparable efficacy to established tools. This concise design in conjunction with a weighted criteria approach facilitates the rapid evaluation of health information for a diverse group of users. For example, health care professionals may use the QUEST to evaluate the quality of information brought to them by their patients or to find high-quality articles to recommend. The QUEST may also be of value to the scientific community as it can be used as a research tool to quickly and accurately evaluate quality, facilitating the characterization and comparison of large amounts of information. Additionally, the QUEST may help inform creators of online health content, including government, industry, university, and advocacy groups, during the content development process.

In terms of content, the QUEST tool is differentiated from the three other tools included in the present analysis in its weighted measurement of tone, conflict of interest, and complementarity (see Table 1). These criteria address factors such as potential bias linked to promotion of a product or intervention, whether support of the patient-physician relationship is referenced, and whether the information is presented in a balanced way.

Finally, the QUEST was designed for application to a variety of health topics including information on both treatment and prevention, as well as general health information. Altogether these characteristics, combined with evidence of the QUEST’s reliability and validity, are reflective of a versatile tool suited to meet diverse user needs. It is important to note that each individual item provides information about only a single aspect of information quality, and thus the QUEST should be used as a gestalt to provide an overall assessment of quality.

It should be noted that while the QUEST is designed to be a concise and universally applicable tool, there is a range of other evaluation tools in the literature with different and potentially complementary aims to QUEST (please see Appendix 2 for a comparison of currently available tools to QUEST). For example, the QIMR tool released in 2017 may be more suited for evaluating health research reports in the lay media and the AGREE instrument may be best suited to evaluating the quality of clinical practice guidelines. While the versatility of the QUEST tool lies in its applicability to a range of online health information, is not necessarily the only or most suitable tool for all types of health-related media.

The focused area of the samples used in this study addresses an important and growing issue relating to the quality of online health information targeted toward aging populations, who face unique challenges in cognition which can be exacerbated by low health literacy [41]. Additionally, older adults tend to have less experience conducting online searches and critically evaluating the credibility of online information [42, 43]. Due to this combination of factors, this demographic of health consumers may be more susceptible to misinformation online. Beyond the focus on AD used for this validation study, the QUEST will benefit from further testing across a wider range of health conditions.

The study design has several strengths. The correlational method used does not rely on an assumption of normality of the data, and the magnitudes of the correlation coefficients indicate the strength of correlation between the tools being compared [39]. We conducted more than one analysis on the data, comparing the QUEST to three well-established and well-regarded evaluation tools. Careful selection of tools for comparison and use of multiple tools in the analysis both contribute to the rigour of the study.

However, we also recognize the limitations of the study. A sample of convenience of a relatively small number of articles was used, taken from existing collections of AD treatment and prevention articles. Due potentially to the small sample size of articles used, the Kendall’s tau scores have substantially overlapping confidence intervals; this indicates a need for further validation studies that include a larger number of articles on other health conditions and on types of health information beyond treatment and prevention, such as descriptions of symptoms and management. Furthermore, our study included only three raters, whereas it may be useful to include more raters in the future when assessing inter-rater reliability. It may also be informative to assess the predictive validity of real-life application of the tool. This may be used to predict whether sustained use of the instrument is associated with higher levels of user knowledge, engagement with care providers on the health topic, or self-efficacy in management of the health condition researched.

Additionally, existing quality evaluation tools generally adopt the perspective of the health care professional in conceptualizing quality [27]. We recognize that the QUEST tool, currently aimed at health care professionals and researchers, falls into this category. Given the time constraints of clinical visits, health care professionals may not be able to assess the quality of online resources during the consultation. To address this issue, attempts have been made to automate tools such as the HONcode and the QUEST [44, 45]. Further, research indicates that the methods used by health consumers to search and appraise online health information differ from the systematic methods used by investigators [46]. As a partially non-academic area of research, a number of health information evaluation tools are not detailed or evaluated in the peer-reviewed literature and may have been excluded from the scoping review presented here. Existing efforts to expand the user base for quality evaluation tools include the HONcode Health Website Evaluation Tool and Provost et al.’s 95-item WebMedQual assessment [47]. This body of work can be expanded upon in the academic space by standardising and ensuring validity of the broad range of heterogeneous tools that exist outside of this space. Future work should continue efforts to develop a more accessible and concise patient-friendly tool that incorporates the values of end-users when assessing online health information, such as privacy and usability factors. To address this need, we are currently in the process of developing a public-friendly adaptation of the existing QUEST criteria that can be easily understood and applied by non-expert users.

Finally, a novel tool aiming to address the issue of misinformation online – whether intended for use by expert or non-expert users – needs to be supplemented by a careful examination of the drivers of public attitudes toward key issues in health care. Studies have shown that social beliefs and attitudes related to a range of health issues (e.g., vaccination uptake [48, 49], health and wellbeing in an ageing population [50], uptake of mental health care [51, 52]) pose significant challenges in obtaining optimal public health outcomes. Tools such as QUEST are designed as downstream interventions that can aid health consumers and providers in differentiating between high- and low-quality information online. It is unlikely that the wide availability of these tools will be effective as a standalone intervention; additional work is required to contextualize the public spaces in which these evaluation tools will be useful and to determine how these tools can best be used in complement to health communication strategies and more upstream, systemic interventions in order to change health behaviours and attitudes.

## Conclusions

Developed to address gaps in available quality evaluation tools for online health information, the QUEST is composed of a short set of criteria that can be used by health care professionals and researchers alike. Our findings demonstrate the QUEST’s reliability and validity in evaluating online articles on AD treatment and prevention. For example, two similar tools used for comparison, the DISCERN and HONcode Health Website Evaluation tools, are 12–16 questions in length. This study provides evidence that the QUEST builds on the strengths of existing instruments and evaluates quality with similar efficacy using a rapid seven-item questionnaire. As a result, this tool may serve as a more accessible resource that effectively consolidates the quality criteria outlined in previous work. Additionally, due to its simplicity and unique weighting approach, the QUEST reduces the need for users’ subjective judgment and indicates potential for future iterations of the tool to be easily tailored to the needs of different users. Based on the current evidence, the QUEST can be used to reliably assess online sources of information on treatment and prevention of AD. Following formal establishment of its reliability and validity across a wide range of health topics, the QUEST may serve as or inform a universal standard for the quality evaluation of online health information.

## Additional files

Additional file 1: Quality scores of treatment articles (*n* = 16). Scores generated by the QUEST, HONcode, Sandvik, and DISCERN tools for the 16 articles containing information on the treatment of AD. (XLSX 57 kb)

Additional file 2: Quality scores of prevention articles (*n* = 29). Scores generated by the QUEST, HONcode, Sandvik, and DISCERN tools for the 29 articles containing information on the prevention of AD. (XLSX 57 kb)

## Appendix 1

**Table 6:** Characteristics of articles (*n* = 36) retrieved between January 15, 2016 and February 5, 2016 using the following search terms on Google Scholar and PubMed: *online, health information, evaluate, evaluation, tool, quality, validity, testing, validation,* and *assessment* and meeting the following inclusion criteria: 1) the article is in the English language; 2) validation of an assessment tool related to quality of health information was the focus of the article

Focus of article	Number of articles	Article title	Author(s)	Date of Publication
Observational or descriptive paper	5	Assessing, controlling, and assuring the quality of medical information on the internet: Caveant lector et viewor—let the reader and viewer beware	Silberg WM, Lundberg GD, and Musacchio RA	1997
		The Health On the Net Code of Conduct for Medical and Health Websites	Boyer, C., M. Selby, J. R. Scherrer, and R. D. Appel	1998
		Emerging Challenges in Using Health Information from the Internet	Theodosiou, Louise, and Jonathan Green	2003
		Health information and the internet: The 5 Cs website evaluation tool	Roberts, Lorraine	2010
		Quality of patient health information on the Internet: reviewing a complex and evolving landscape	Fahy, Eamonn, Rohan Hardikar, Adrian Fox, and Sean Mackay	2014
Evaluation of quality of information using tool(s)	7	Health information and interaction on the internet: a survey of female urinary incontinence	Sandvik, Hogne	1999
		Evaluation of Websites that Provide Information on Alzheimer’s Disease	Bouchier, H., and P. A. Bath	2003
		Accuracy of internet recommendations for prehospital care of venomous snake bites	Barker et al	2010
		The quality of online antidepressant drug information: An evaluation of English and Finnish language Web sites	Prusti, Marjo, Susanna Lehtineva, Marika Pohjanoksa-Mäntylä, and J. Simon Bell	2012
		Evaluation of dengue-related health information on the Internet	Rao et al	2012
		A Methodology to Analyze the Quality of Health Information on the Internet The Example of Diabetic Neuropathy	Chumber, Sundeep, Jörg Huber, and Pietro Ghezzi	2014
		Evaluation of Online Health Information on Clubfoot Using the DISCERN Tool	Kumar, Venkatesan S., Suresh Subramani, Senthil Veerapan, and Shah A. Khan	2014
Development of tool	12	DISCERN: an instrument for judging the quality of written consumer health information on treatment choices.	Charnock, D., S. Shepperd, G. Needham, and R. Gann	1999
		Development of a self-assessment method for patients to evaluate health information on the Internet	Jones J.	1999
		Development and Application of a Tool Designed to Evaluate Web Sites Providing Information on Alzheimer’s Disease	Bath, P. A., and H. Bouchier	2003
		Development and validation of an international appraisal instrument for assessing the quality of clinical practice guidelines: the AGREE project	Cluzeau et al.	2003
		Design and testing of a tool for evaluating the quality of diabetes consumer-information web sites. Journal of Medical Internet Research	Seidman, Joshua J, Donald Steinwachs, and Haya R Rubin	2003
		The development of QUADAS: a tool for the quality assessment of studies of diagnostic accuracy included in systematic reviews	Whiting, Penny, Anne WS Rutjes, Johannes B. Reitsma, Patrick MM Bossuyt, and Jos Kleijnen	2003
		Ensuring Quality Information for Patients: Development and Preliminary Validation of a New Instrument to Improve the Quality of Written Health Care Information	Moult, Beki, Linda S Franck, and Helen Brady	2004
		A model for online consumer health information quality	Stvilia, Besiki, Lorri Mon, and Yong Jeong Yi	2009
		Health Literacy INDEX: Development, Reliability, and Validity of a New Tool for Evaluating the Health Literacy Demands of Health Information Materials	Kaphingst et al.	2012
		Measuring the quality of Patients’ goals and action plans: development and validation of a novel tool	Teal, Cayla R., Paul Haidet, Ajay S. Balasubramanyam, Elisa Rodriguez, and Aanand D. Naik	2012
		The Communication AssessmenT Checklist in Health (CATCH): a tool for assessing the quality of printed educational materials for clinicians.	Genova, Juliana, Isaac Nahon-Serfaty, Selma Chipenda Dansokho, Marie-Pierre Gagnon, Jean-Sébastien Renaud, and Anik M. C. Giguère	2014
		Development and Validation of the Guide for Effective Nutrition Interventions and Education (GENIE): A Tool for Assessing the Quality of Proposed Nutrition Education Programs	Hand, Rosa K., Jenica K. Abram, Katie Brown, Paula J. Ziegler, J. Scott Parrott, and Alison L. Steiber	2015
Evaluation of tool(s)	9	Published Criteria for Evaluating Health Related Web Sites: Review	Kim, Paul, Thomas R. Eng, Mary Jo Deering, and Andrew Maxfield	1999
		Examination of instruments used to rate quality of health information on the internet: chronicle of a voyage with an unclear destination	Gagliardi, Anna, and Alejandro R. Jadad	2002
		Evaluating the reliability and validity of three tools to assess the quality of health information on the Internet.	Ademiluyi, Gbogboade, Charlotte E Rees, and Charlotte E Sheard	2003
		The Evaluation Criteria of Internet Health Information	Kang, Nam-Mi, Sukhwa Kim, Seungkuen Hong, Seewon Ryu, Hye-Jung Chang, and Jeongeun Kim	2006
		Assessing the Quality of Websites Providing Information on Multiple Sclerosis: Evaluating Tools and Comparing Sites	Harland, Juliet, and Peter Bath	2007
		What Do Evaluation Instruments Tell Us About the Quality of Complementary Medicine Information on the Internet?	Breckons, Matthew, Ray Jones, Jenny Morris, and Janet Richardson	2008
		Tools Used to Evaluate Written Medicine and Health Information Document and User Perspectives	Luk, Alice, and Parisa Aslani	2011
		Tools for Assessing the Quality and Accessibility of Online Health Information: Initial Testing among Breast Cancer Websites	Whitten, Pamela, Samantha Nazione, and Carolyn Lauckner	2013
		Web-site evaluation tools: a case study in reproductive health information	Aslani, Azam, Omid Pournik, Ameen Abu-Hanna, and Saeid Eslami	2014
Systematic literature review of tools	3	Empirical Studies Assessing the Quality of Health Information for Consumers on the World Wide Web: A Systematic Review	Eysenbach et al.	2002
		Online Health Information Tool Effectiveness for Older Patients: A Systematic Review of the Literature	Bolle, Sifra, Julia C. M. van Weert, Joost G. Daams, Eugène F. Loos, Hanneke C. J. M. de Haes, and Ellen M. A. Smets.	2015
		Quality of Health Information for Consumers on the Web: A Systematic Review of Indicators, Criteria, Tools, and Evaluation Results	Zhang, Yan, Yalin Sun, and Bo Xie	2015

## Appendix 2

**Table 7:** Comparison of evaluation tools previously described in the literature and QUEST

	Name of tool	Focus	Criteria	Format
0	QUality Evaluation Scoring Tool (QUEST)	Quality of online health information	Authorship, attribution, conflict of interest, complementarity, currency, tone	6 questions rated on a scale of 0–2 or 0–1 and differentially weighted, yielding an overall quality score between 0 and 28
1	DISCERN	Quality of written information about treatment choices	Reliability, balance, dates, source, quality of information on treatment sources, overall rating	15 questions rated on a scale of 1–5
2	EQIP: Ensuring Quality Information for Patients	Quality of written patient information applicable to all information types	Clarity, patient-oriented design, currency, attributon, conflict of interest, completeness	20 questions rated Y/Partly/N with an equation to generate a % score
3	Jones’ Self-Assessment Method	Self-assessment tool for patients to evaluate quality and relevance of health care oriented websites	Content, design, communication, and credibility	9 broad questions based on 4 criteria rated Yes/No/NA
4	Health on the Net Foundation’s HONcode Patient Evaluation Tool	Patient evaluation tool for health-related websites	Authorship, attribution, currency, reliability, balance, mission/target audience, privacy, interactivity, overall reliability	16-item interactive questionnaire returning a % score
5	Silberg standards	Standards of quality for online medical information for consumers and professionals	Authorship, attribution, disclosure, currency	Set of core standards; no score is generated
6	Sandvik’s General Quality Criteria	General quality measure for online health information	Ownership, authorship, source, currency, interactivity, navigability, balance	7 questions rated on a scale of 0–2
7	Health Information Technology Institute (HITI) Information Quality Tool **No longer available*	Quality measure for health-related websites	Credibility, content, disclosure, links, design, interactivity	*Not available*
8	5 C’s website evaluation tool	Structured guide to systematically evaluating websites; specifically developed for nurses to use in patient care and education	Credibility, currency, content, construction, clarity	Series of 36 open-ended and yes/no questions grouped under the “5 C’s”; no score is generated
9	Health Literacy INDEX	Tool to evaluate the health literacy demands of health information materials	Plain language, clear purpose, supporting graphics, user involvement, skill-based learning, audience appropriateness, instructions, development details, evaluation methods, strength of evidence	63 indicators/criteria rated yes/no, yielding criterion-specific scores and an overall % score
10	Bath and Bouchier’s evaluation tool	Tool to evaluate websites providing information on Alzheimer’s disease	General details, information for carers, currency, ease of use, general conclusions	47 questions scored from 0 to 2, generating an overall % score
11	Seidman quality evaluation tool	Quality of diabetes consumer-information websites	Explanation of methods, validity of methods, currency, comprehensiveness, accuracy	7 structural measures and 34 performance measures, generating composite scores by section and an overall score
12	Appraisal of Guidelines, REsearch and Evaluation (AGREE) Collaboration instrument	Quality of clinical practice guidelines	Scope and purpose, stakeholder involvement, rigour of development, clarity and presentation, applicability, editorial independence	23 items grouped into six quality domains with a 4 point Likert scale to score each item
13	Communication AssessmenT Checklist in Health (CATCH) tool	Quality of printed educational materials for clinicians	Appearance, layout and typography, clarity of content, language and readability, graphics, risk communication, scientific value, emotional appeal, relevance, social value/source credibility, social value/usefulness for the clinician, social value/usefulness for the health care system (hospital or government)	55 items nested in 12 concepts, each rated yes/no, generating concept-specific and overall scores
14	LIDA Minervation tool	Evaluates the design and content of healthcare websites	Accessibility, usability (clarity, consistency, functionality, engagability), reliability (currency, conflict of interest, content production)	41 questions scored on a scale of 0–3, yielding a total % score
15	Mitretek Information Quality Tool (IQT) **no longer available*	Evaluates information quality of online health information	Authorship, sponsorship, currency, accuracy, confidentiality, navigability	21 questions rated yes/no and weighted according to importance, generating a total score between 0 to 4
16	“Date, Author, References, Type, and Sponsor” (DARTS)	Assists patients in appraising the quality of online medicines information	Currency, authorship, credibility, purpose, conflict of interest	A series of six guiding questions; no score generated
17	Quality Index for health-related Media Reports (QIMR)	Monitors the quality of health research reports in the lay media	Background, sources, results, context, validity	17 items rated on a 0–6 Likert scale with an 18th global rating
18	Index of Scientific Quality (ISQ)	Index of scientific quality for health reports in the lay press	Applicability, opinions vs. facts, validity, magnitude, precision, consistency, consequences	7 items rated on a 1–5 Likert scale with an 8th global rating

## Abbreviations

AD: Alzheimer disease
CI: Confidence interval
QUEST: QUality Evaluation Scoring Tool

## Footnotes

1 Please refer to Appendix 1 for characteristics of retrieved articles.

2 Please refer to Appendix 2 for a complete listing of currently available tools.

## References

[1] Fox S. Health topics: Pew Research Center: Internet, Science & Tech; 2011. http://www.pewinternet.org/2011/02/01/health-topics-2/. Accessed 21 Apr 2016

[2] Stvilia B, Mon L, Yi YJ (2009). A model for online consumer health information quality. J Am Soc Inf Sci Technol.

[3] Theodosiou L, Green J (2003). Emerging challenges in using health information from the internet. Adv Psychiatr Treat.

[4] McCully SN, Don BP, Updegraff JA (2013). Using the internet to help with diet, weight, and physical activity: results from the health information National Trends Survey (HINTS). J Med Internet Res.

[5] Fahy E, Hardikar R, Fox A, Mackay S (2014). Quality of patient health information on the internet: reviewing a complex and evolving landscape. Australas Med J.

[6] Rootman I, Gordon-El-Bihbety D. A vision for a health literate Canada: Canadian public health association; 2008. https://www.cpha.ca/vision-health-literate-canada-report-expert-panel-health-literacy. Accessed 22 Aug 2016.

[7] Kutner M, Greenberg E, Jin Y, Paulsen C. The health literacy of America’s adults: results from the 2003 National Assessment of adult literacy: National Center for Education Statistics; 2006. https://nces.ed.gov/pubsearch/pubsinfo.asp?pubid=2006483. Accessed 22 Aug 2016.

[8] Diviani Nicola, van den Putte Bas, Giani Stefano, van Weert Julia CM (2015). Low Health Literacy and Evaluation of Online Health Information: A Systematic Review of the Literature. Journal of Medical Internet Research.

[9] Devine Theresa, Broderick Jordan, Harris Linda M, Wu Huijuan, Hilfiker Sandra Williams (2016). Making Quality Health Websites a National Public Health Priority: Toward Quality Standards. Journal of Medical Internet Research.

[10] Arksey H, O’Malley L (2005). Scoping studies: towards a methodological framework. Int J Soc Res Methodol.

[11] Zeraatkar D, Obeda M, Ginsberg JS, Hirsh J (2017). The development and validation of an instrument to measure the quality of health research reports in the lay media. BMC Public Health.

[12] Närhi U, Pohjanoksa-Mäntylä M, Karjalainen A, Saari JK, Wahlroos H, Airaksinen MS (2008). The DARTS tool for assessing online medicines information. Pharm World Sci.

[13] Oxman AD, Guyatt GH, Cook DJ, Jaeschke R, Heddle N, Keller J (1993). An index of scientific quality for health reports in the lay press. J Clin Epidemiol.

[14] Hsu W-C, Bath PA (2008). Development of a patient-oriented tool for evaluating the quality of breast cancer information on the internet. Stud Health Technol Inform..

[15] Seidman JJ, Steinwachs D, Rubin HR (2003). Design and testing of a tool for evaluating the quality of diabetes consumer-information web sites. J Med Internet Res.

[16] Charnock D, Shepperd S, Needham G, Gann R (1999). DISCERN: an instrument for judging the quality of written consumer health information on treatment choices. J Epidemiol Community Health.

[17] Moult B, Franck LS, Brady H (2004). Ensuring quality information for patients: development and preliminary validation of a new instrument to improve the quality of written health care information. Health Expect Int J Public Particip Health Care Health Policy.

[18] Jones J. Development of a self-assessment method for patients to evaluate health information on the Internet. Proc AMIA Symp. 1999:540–4. PMC2232549 10566417

[19] Aslani A, Pournik O, Abu-Hanna A, Eslami S (2014). Web-site evaluation tools: a case study in reproductive health information. Stud Health Technol Inform.

[20] Eysenbach G, Powell J, Kuss O, Sa E-R (2002). Empirical studies assessing the quality of health information for consumers on the world wide web: a systematic review. JAMA.

[21] Kim P, Eng TR, Deering MJ, Maxfield A (1999). Published criteria for evaluating health related web sites: review. BMJ.

[22] Roberts L (2010). Health information and the internet: the 5 Cs website evaluation tool. Br J Nurs.

[23] Breckons M, Jones R, Morris J, Richardson J (2008). What do evaluation instruments tell us about the quality of complementary medicine information on the internet?. J Med Internet Res.

[24] Bernstam EV, Shelton DM, Walji M, Meric-Bernstam F (2005). Instruments to assess the quality of health information on the world wide web: what can our patients actually use?. Int J Med Inf.

[25] Gagliardi A, Jadad AR (2002). Examination of instruments used to rate quality of health information on the internet: chronicle of a voyage with an unclear destination. BMJ.

[26] Prusti M, Lehtineva S, Pohjanoksa-Mäntylä M, Bell JS (2012). The quality of online antidepressant drug information: an evaluation of English and Finnish language web sites. Res Soc Adm Pharm.

[27] Ademiluyi G, Rees CE, Sheard CE (2003). Evaluating the reliability and validity of three tools to assess the quality of health information on the internet. Patient Educ Couns.

[28] Sandvik H (1999). Health information and interaction on the internet: a survey of female urinary incontinence. BMJ.

[29] Chumber S, Huber J, Ghezzi P (2015). A methodology to analyze the quality of health information on the internet: the example of diabetic neuropathy. Diabetes Educ.

[30] Silberg W, Lundberg G, Musacchio R (1997). Assessing, controlling, and assuring the quality of medical information on the internet: caveant lector et viewor—let the reader and viewer beware. JAMA.

[31] Robillard JM, Feng TL (2017). Health advice in a digital world: quality and content of online information about the prevention of Alzheimer’s disease. J Alzheimers Dis.

[32] What is GRADE? BMJ Clinical Evidence. 2012. http://clinicalevidence.bmj.com/x/set/static/ebm/learn/665072.html. Accessed 14 Feb 2017.

[33] Robillard JM, Johnson TW, Hennessey C, Beattie BL, Illes J (2013). Aging 2.0: health information about dementia on twitter. PLoS One.

[34] Robillard JM, Illes J, Arcand M, Beattie BL, Hayden S, Lawrence P (2015). Scientific and ethical features of English-language online tests for Alzheimer’s disease. Alzheimers Dement Diagn Assess Dis Monit.

[35] Beitzel SM, Jensen EC, Chowdhury A, Frieder O, Grossman D (2007). Temporal analysis of a very large topically categorized web query log. J Am Soc Inf Sci Technol.

[36] Bouchier H, Bath PA (2003). Evaluation of websites that provide information on Alzheimer’s disease. Health Informatics J..

[37] Harland J, Bath P (2007). Assessing the quality of websites providing information on multiple sclerosis: evaluating tools and comparing sites. Health Informatics J.

[38] Landis JR, Koch GG (1977). The measurement of observer agreement for categorical data. Biometrics.

[39] Gibbons JD (1985). Nonparametric statistical inference.

[40] Health Website Evaluation Tool. http://www.hon.ch/HONcode/Patients/HealthEvaluationTool.html. Accessed 11 Apr 2016.

[41] Mullen E (2013). Health literacy challenges in the aging population. Nurs Forum (Auckl).

[42] Robertson-Lang L, Major S, Hemming H (2011). An exploration of search patterns and credibility issues among older adults seeking online health information. Can J Aging Rev Can Vieil.

[43] Kruse RL, Koopman RJ, Wakefield BJ, Wakefield DS, Keplinger LE, Canfield SM (2012). Internet use by primary care patients: where is the digital divide?. Fam Med.

[44] Boyer C, Dolamic L (2015). Automated detection of HONcode website conformity compared to manual detection: an evaluation. J Med Internet Res.

[45] Robillard JM, Alhothali A, Varma S, Hoey J (2017). Intelligent and affectively aligned evaluation of online health information for older adults. Workshops at the thirty-first AAAI conference on artificial intelligence.

[46] Eysenbach G, Köhler C (2002). How do consumers search for and appraise health information on the world wide web? Qualitative study using focus groups, usability tests, and in-depth interviews. BMJ.

[47] Provost M, Koompalum D, Dong D, Martin BC (2006). The initial development of the WebMedQual scale: domain assessment of the construct of quality of health web sites. Int J Med Inf..

[48] Fournet N, Mollema L, Ruijs WL, Harmsen IA, Keck F, Durand JY, et al. Under-vaccinated groups in Europe and their beliefs, attitudes and reasons for non-vaccination; two systematic reviews. BMC Public Health Lond. 2018;18. 10.1186/s12889-018-5103-8. 10.1186/s12889-018-5103-8 PMC5789742 29378545

[49] Larson HJ, Jarrett C, Schulz WS, Chaudhuri M, Zhou Y, Dube E (2015). Measuring vaccine hesitancy: the development of a survey tool. Vaccine.

[50] Robertson G (2016). Attitudes towards ageing and their impact on health and wellbeing in later life: an agenda for further analysis. Work Older People.

[51] Prins Marijn A., Verhaak Peter F.M., Bensing Jozien M., van der Meer Klaas (2008). Health beliefs and perceived need for mental health care of anxiety and depression—The patients' perspective explored. Clinical Psychology Review.

[52] Coates D, Saleeba C, Howe D. Mental health attitudes and beliefs in a community sample on the central coast in Australia: barriers to help seeking. Community Ment Health J. 2018. 10.1007/s10597-018-0270-8. 10.1007/s10597-018-0270-8 29589218

